# Tailoring polymethylhydrosiloxane as candidate material for vitreous humour substitution: physical properties and in vitro toxicity

**DOI:** 10.1186/s13065-025-01548-5

**Published:** 2025-07-11

**Authors:** Diba G. Auliya, Vira F. Arini, Tiara Nurmadanti, Ulfa Fauziah, Soni Setiadji, Fitrilawati Fitrilawati, Risdiana Risdiana

**Affiliations:** 1 Doctor Program in Biotechnology, Graduate School, Universitas Padjadjaran, Bandung, Indonesia; 2 Department of Physics, Faculty of Mathematics and Natural Sciences, Universitas Padjadjaran, Sumedang, Indonesia; 3 Department of Chemistry, Faculty of Science and Technology, UIN Sunan Gunung Djati, Bandung, Indonesia

**Keywords:** Dichloromethylsilane, In vitro toxicity, Physical properties, Polymethylhydrosiloxane, Vitreous humour

## Abstract

Polymethylhydrosiloxane (PMHS) has been considered to be developed as an alternative material of polydimethylsiloxane (PDMS) for vitreous humour substitution. This polymer production begins with hydrolysis of dichloromethylsilane (DCMS), as raw material, which continues through condensation polymerization. Previous research reported the synthesis of PMHS using an acid solvent with different temperature variations and indicated that low-viscosity PMHS can be produced through condensation at 15–20 °C. However, this process requires a very long polymerization time. Meanwhile, synthesis using a higher temperature of 50 °C required a catalyst. The influence of solvents, one of the important synthesis temperatures, on the synthesis process of PMHS has not been explored. Furthermore, the toxicity of this material has not been reported. In this study, PMHS with low- and medium-viscosity were synthesized from DCMS with different solvents and additional control of the condensation temperature to accelerate the polymerization. Utilizing a basic diethyl ether (DE) solvent facilitates a higher viscosity value than an acidic dichloromethane (DCM) solvent. All PMHS samples were characterized by a viscometer, refractometer, surfgauge, UV–Vis, FTIR, and NMR spectroscopy. The in vitro hen’s egg chorioallantoic membrane (HET-CAM) toxicity test of PMHS was also conducted. A low-viscosity PMHS sample had a surface tension of 20 milliN/m and a refractive index of 1.3960, while medium-viscosity had 21 milliN/m and 1.3982. All samples were ~ 100% transparent, had typical functional groups of PMHS, and did not show signs of being irritant (non-toxic). Therefore, PMHS has the potential to be developed as a new material for vitreous humour substitution.

## Introduction

Retinal detachment is a vitreous humour disorder that can cause blindness. In this condition, the retinal neurosensory segment is released from the retinal epithelium [1, 2]. Silicone oil or polydimethylsiloxane (PDMS) is recommended in difficult cases of surgical treatment to treat retinal detachment [3–5]. It is known as the substitution fluid for vitreous humour to push the separated retina to its natural position and rebuild the volume of the vitreous cavity.

The materials used as a vitreous substitute must have properties similar to natural vitreous, transparent, biocompatible for long-term use, stable and easy to store, non-toxic, can be put into a small syringe, and available at a reasonable price. This material must also be able to be synthesized effectively and be produced on a large scale [1, 2, 6]. Therefore, various characterizations and tests are needed to obtain information on the characteristics and formulation of materials.

Due to the increasing cases of retinal detachment, the need for PDMS as a substitute for vitreous humour has also increased. PDMS is hydrophobic, has stable physicochemical properties for short and long-term use, and has low toxicity. In its use as a substitute for vitreous humor, PDMS has disadvantages in emulsification which can result in complications [1, 2, 6]. PDMS is commonly synthesized from octamethylcyclotetrasiloxane (D4) by ring-opening polymerization (ROP) procedure [7–10]. It can also be synthesized from silane materials by hydrolysis-condensation method [11–14]. In the polymerization process, it is tough to avoid the reformation of D4. It has been reported that the presence of D4 content affects both the stability and toxicity of PDMS materials [15]. Furthermore, the difficulty in accessing these materials presents a significant challenge in synthesizing PDMS. Therefore, alternative materials that are more efficient for synthesizing vitreous substitutes need to be developed.

Polymethylhydrosiloxane (PMHS), a useful polysiloxane for the preparation of high-value silicone material, is a polymer that consists of an inorganic Si–O backbond, pendant organic methyl group, and hydrogen [16, 17]. PMHS is a colorless free-flowing liquid that has the advantage of being steady and non-toxic. Therefore, it can be used as an alternative to PDMS. However, commercial PMHS has a low viscosity of ~ 12 to 45 mPa.s [18]. As vitreous humour substitution, PMHS must have a viscosity of at least 900 mPa.s and a maximum of 5500 mPa.s [3, 7].

In previous investigations, PMHS with suitable viscosity for vitreous humour substitution was obtained from dichloromethylsilane (DCMS) (CH_3_HSiCl_2_), which is more accessible than D4. DCMS with dichloromethane (DCM) as solvent (rasio 1:4) will produce siloxane material through the hydrolysis process, followed by polymerization via condensation at 15–20 °C to form PMHS [19]. However, this condensation method requires a long polymerization time (72 days) for the sample to reach a viscosity that meets vitreous substitute standards. Using the same material with a smaller volume ratio of 1:3, PMHS with a viscosity of 930 mPa.s has been successfully synthesized at a temperature of 50 °C by adding heating treatment and KOH as a catalyst in the polymerization process [20]. Meanwhile, a higher viscosity category has not yet been obtained. Therefore, other techniques to accelerate the polymerization process need to be explored.

The solvent is one of the important parameters for synthesizing long-chain polysiloxane materials with a certain viscosity using the hydrolysis-condensation method. The solvent used can be an acid or a base [12, 16, 21]. Therefore, the effect of solvent on viscosity needs to be explored. Furthermore, a toxicity assessment should also be conducted to evaluate the performance and safety of PMHS as vitreous humour substitution. In this study, two viscosity types of PMHS will be synthesized using different solvents, diethyl ether (DE) and dichloromethane (DCM). The polymerization process will be accelerated by controlling the condensation temperature. The physical properties of the PMHS such as viscosity, refractive index, surface tension, and transparency will be investigated along with the functional groups of the material. The toxicity of the material will be assessed through the hen’s egg chorioallantoic membrane (HET-CAM) test. This toxicity test is an in vitro test that observes the presence of haemorrhage, coagulation, and lysis as the endpoints in hen’s eggs membrane which similar to rabbit eye tissue [22].

## Materials and method

PDMS is a linear polysiloxane containing repeating units of the formula [(CH_3_)_2_SiO]. Meanwhile, the end-blocking units of PDMS consist of (CH_3_)_3_SiO. In this research, commercial PDMS 1300 (ARCIOLANE) from Arcadoptha is presented for reference and comparison for the vitreous substitute material [8].

PMHS, the alternative of PDMS, was synthesized by the hydrolysis-condensation method. The chemical reaction for the synthesis of PMHS by this method is shown in Eq. (1).

$${\text{n CH}}_{{3}} {\text{SiHCl}}_{{2}} \, + \,{\text{2n H}}_{{2}} {\text{O}}\, \to \,{\text{n CH}}_{{3}} {\text{SiH}}\left( {{\text{OH}}} \right)_{{2}} + {\text{ 2n HCl}}$$

1 $${\text{n CH}}_{{3}} {\text{SiH}}\left( {{\text{OH}}} \right)_{{2}} + {\text{ CH}}_{{3}} {\text{SiH}}\left( {{\text{OH}}} \right)_{{2}} \, \to \,{\text{OH}} - \left( { - {\text{CH}}_{{3}} \left( {\text{H}} \right){\text{SiO}} - } \right)_{{{\text{n}} + {1}}} - {\text{H }} + {\text{ n H}}_{{2}} {\text{O}}$$

Two PMHS samples with different viscosities were synthesized using different solvents in the hydrolysis process. Medium-viscosity PMHS was synthesized using diethyl ether (DE) as the solvent, while low-viscosity PMHS was synthesized using dichloromethane (DCM) solvent. The reaction began by mixing 25 mL of DCMS, 25 mL of solvent, and 12,5 mL milli-Q water at 35 °C for 3 h. After this process, the solvent was evaporated. Then, the condensation process was carried out by stirring the sample by controlling the condensation temperature at 35 °C for a certain time. To remove unwanted residuals, a purification process was carried out by adding chloroform and milli-Q water as written in the previous study [16]. Medium-viscosity PMHS was addressed as A-1, while low-viscosity PMHS as A-2. The synthesis conditions of the samples are shown in Table 1.

**Table 1:** Synthesis parameters of the PMHS samples

Condition	A-1	A-2
Solvent	DE	DCM
Volume ratio DCMS:solvent	1:1	1:1
Condensation temperature (°C)	35	35
Condensation time (hours)	2	24

The characterization of all samples was performed using Sekonic Viscomate VM-10A-MH digital viscometer to measure viscosity, Handheld AS ONE model I-500 (Brix 0–90%) refractometer to measure refractive index, capillary rise method dyne gauge (DG-1) surfgauge to measure surface tension, PG Instruments Ltd. model T + 70 UV–Vis spectrometer to measure transmittance, and Perkin Elmer Spectrum 100 FTIR spectrometer to identify functional groups. ^1^H-NMR characterization of the samples was also performed using 500 MHz Agilent-VNMRS500 to determine their chemical content.

**Fig. 1:**
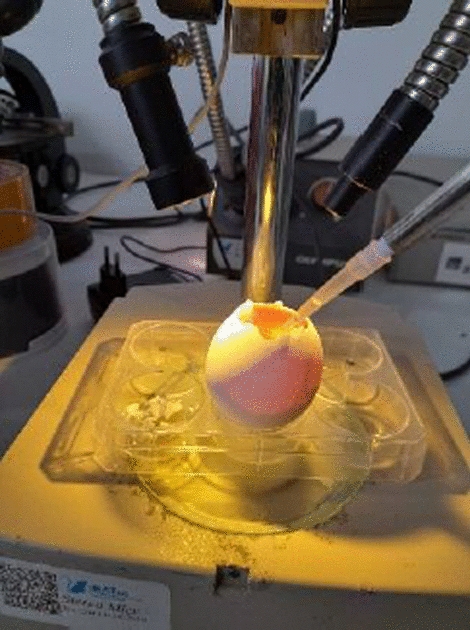
The administration of test material to the egg membrane

The toxicity test of the material was carried out using the HET-CAM Test. The HET-CAM test was performed on fertile white leghorn eggs. The test material used 7-day-old fertile eggs, a positive control of SDS 1% (irritating), and a negative control of NaCl 0.9% (not irritating). Each materials test group contained three eggs. After the eggs were incubated at 37–38 °C for 3 days, 300 μL of the material tests were applied to the chorioallantoic membrane as shown in Fig. 1. Observations were carried out for 300 s by observing the presence of haemorrhage, coagulation, and lysis at 0, 15, 30, 60, 100, and 300 s. The physical and chemical characterizations were also performed on the commercial PDMS for comparative analysis. In its application, the commercial PDMS 1300 utilized is classified as a low-viscosity type.

## Results and discussions

The value of viscosity (η), surface tension ($\gamma$), refractive index (n), and additional diopters of PMHS samples are shown in Table 2. Sample A-1 with a viscosity of 2.10 Pa.s was categorized in the medium viscosity range, while A-2 with a viscosity of 1.10 Pa.s was in the low viscosity range of vitreous humour substitution material. Based on its use, commercial PDMS is categorized as low viscosity if it has a viscosity in the range of 900–1200 mPas. Medium viscosity PDMS has a viscosity in the range of 2000–2400 mPas [23]. Low-viscosity PDMS is easy to inject but easily emulsifies. Meanwhile, PDMS with medium viscosity is easy to inject and has better emulsification resistance than low viscosity [23, 24].

**Table 2:** Characteristics results of polymer samples

Sample	Solvent	η (Pa.s)	$\gamma$ (milliN/m)	n	Additional diopters
A-1	DE	2.10	21	1.3982	3.120
A-2	DCM	1.10	20	1.3960	3.016
Commercial PDMS [8]	–	1.08	20	1.4044	3.430

The solvent used and the synthesis parameters affected the viscosity of both samples. Hydrolysis conducted under basic conditions is reported to yield samples with a higher molecular weight compared to hydrolysis performed under acidic conditions [25]. A basic solvent will neutralize the HCl by-product, thereby preventing conditions from becoming acidic which can slow down the reaction. Furthermore, basic conditions enhance silanol group deprotonation, making it more reactive and accelerating the condensation reaction. Therefore, polymerization becomes faster and produces a sample with a high molecular weight [26]. Since molecular weight is directly proportional to the viscosity, this implies that the viscosity value obtained using basic DE as the solvent will be higher than using acidic DCM. In addition, elevating the temperature during the condensation process accelerates the polymerization process compared to the polymerization conducted under condensation with lower temperature (15–20 °C) [19]. Higher temperatures will increase the polymerization rate and resulting higher viscosity in shorter reaction times [26, 27]. This study also shows that the use of basic solvents with the minimum volume ratio (1:1) of DCMS:DCM can reduce the need for solvents and a higher synthesis temperature (50 °C) to accelerate the polymerization process as in previous research [20].

The surface tension of sample A-1 (21 milliN/m) is higher than sample A-2 and commercial PDMS (20 milliN/m). This result proves that surface tension is related to the viscosity. A sample with a large viscosity value indicates that it is formed from a long-chain polymer. As a result, the sample has strong intermolecular bonds which is indicated by a high surface tension value. Surface tension maintains the tension between the surface and subretinal fluid in the eye cavity. It affects the sample's ability to close the retinal tear and prevent fluid from migrating into the subretinal space [4]. Furthermore, the higher the surface tension value, the better the sample quality due to the ability to decrease the emulsification possibility. The surface tension significantly influences the performance of the sample and compatibility with the surrounding ocular environment [28].

PMHS samples have refractive index values from 1.3960 to 1.3982. All refractive index values are lower than the commercial PDMS. The additional diopters were still within the range of the vitreous humour substitution requirement (+ 3.0D until + 3.5D). The additional diopters range of commercial PDMS is from + 3.055D to + 3.430D [29].

**Fig. 2:**
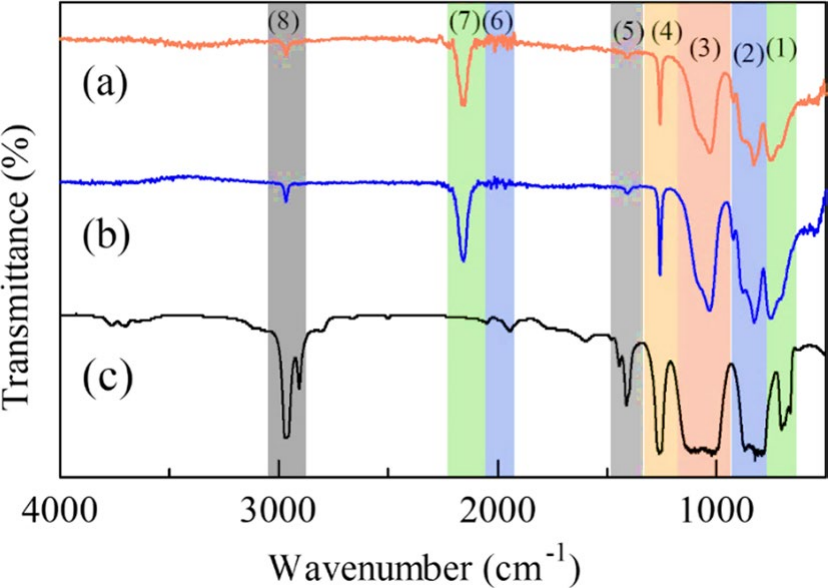
IR Spectra of **a** A-1, **b** A-2, and **c** commercial PDMS

Figure 2 shows the IR spectra of all samples including the commercial PDMS as the comparison. All the samples had exhibited the PMHS functional group, characterized by the presence of Si-O-Si, Si-CH₃, Si–C, C-H, and Si–H bonds [16]. This result had a slight difference with PDMS which did not have the functional group of Si–H as shown in Table 3.

**Table 3:** Functional groups of all samples, commercial PDMS, and literature PMHS

No	Functional group	Wavenumber (cm^−1^)			
		Commercial PDMS [8]	Literature PMHS [16]	A-1	A-2
(1)	Si–O-Si	500, 703	–	754	752
(2)	Si–C stretching and CH_3_ rocking	792–823	750–860	828	828
(3)	Si–O-Si stretching	1022, 1111	1000–1100	1032	1031
(4)	CH_3_ symmetric deformation of Si-CH_3_	1263	1365–1390	1258	1258
(5)	CH_3_ asymmetric deformation of Si-CH_3_	1412	1420–1470	1408	1405
(6)	Si–C	1945, 2052	–	2041	2007
(7)	Si–H	–	2100–2300	2161	2158
(8)	C-H	2905, 2971	2950–2975	2965	2966

**Fig. 3:**
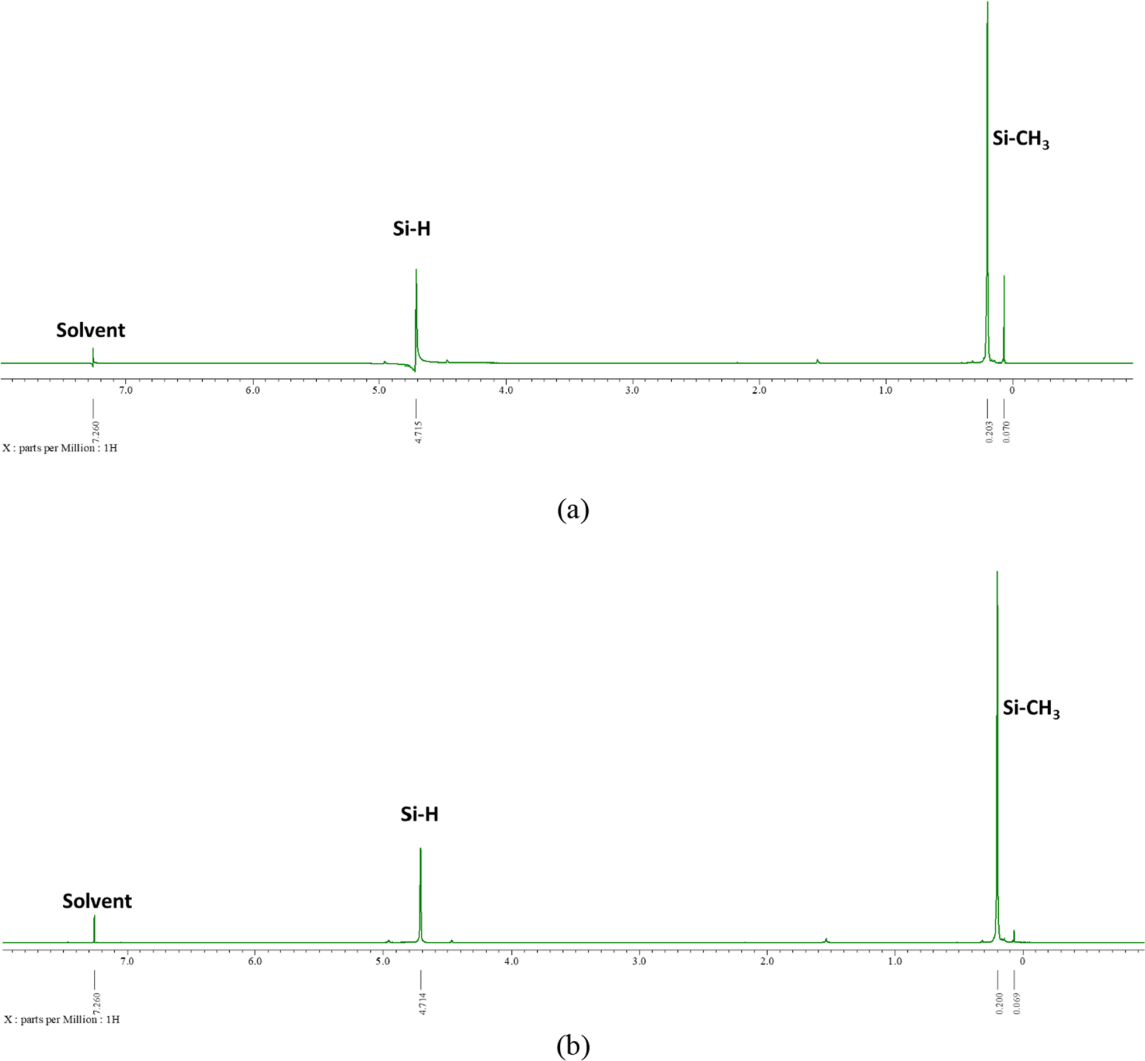
NMR Spectra of sample **a** A-1 and **b** A-2

NMR characterization results in Fig. 3 also confirm that the sample had PMHS characteristics. It is indicated by the presence of peaks that belong to H content in Si–H and Si-CH_3_. Sample A had a Si–H peak at 4.715 ppm and Si-CH_3_ at 0.203 ppm and 0.070 ppm. While sample B had a Si–H peak at 4.714 ppm and Si-CH_3_ at 0.200 ppm and 0.069 ppm.

**Fig. 4:**
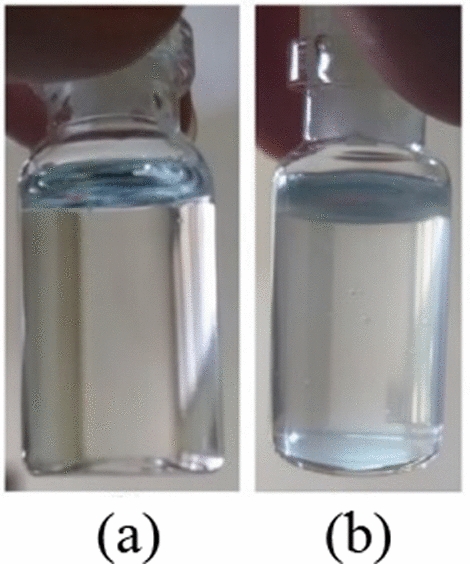
Physical appearance PMHS samples of **a** A-1 and **b** A-2

**Fig. 5:**
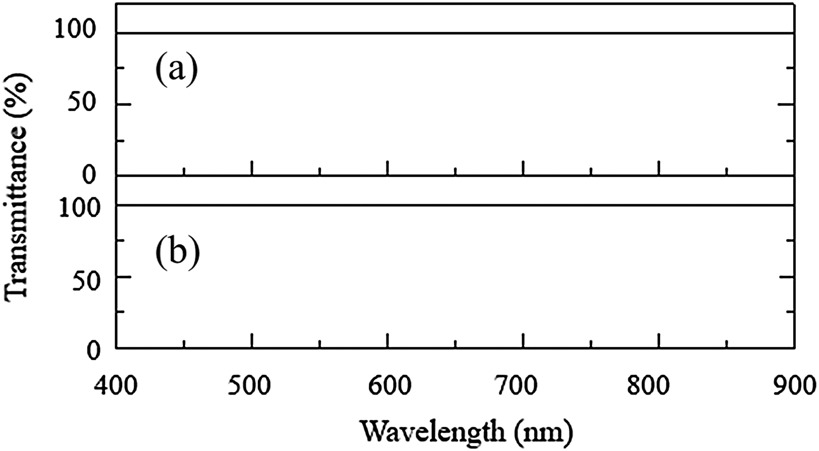
Transmittance in the visible wavelength range of **a** A-1 and **b** A-2

Figure 4 shows the visual appearance of PMHS samples. All samples had a transparent physical appearance. UV–Vis characterization results showed that samples had transmittance values of ~ 100% as shown in Fig. 5. The transmittance of all samples showed that the PMHS samples had similar characteristics as the vitreous substitution.

**Fig. 6:**
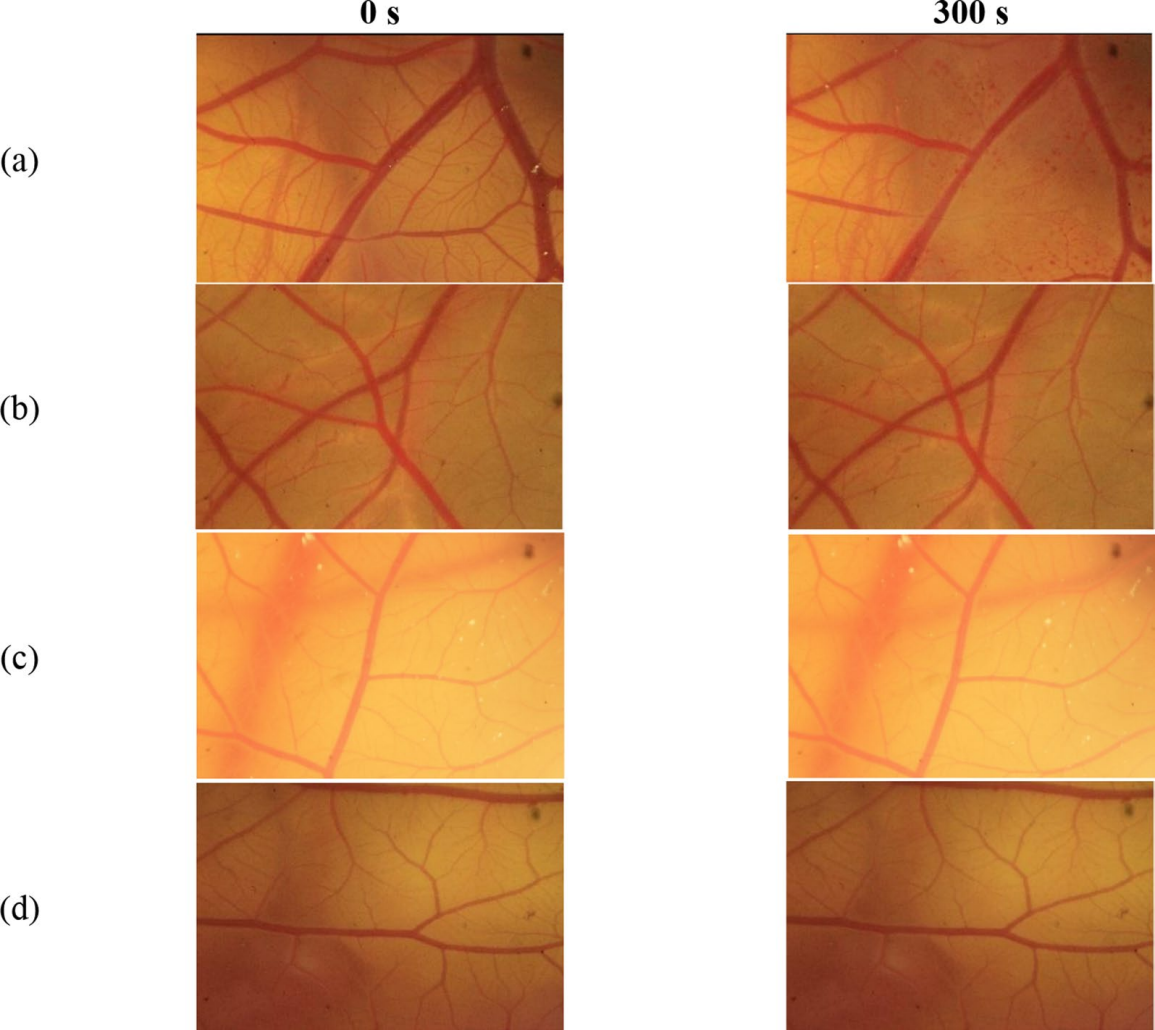
The vessels of **a** SDS (positive control), **b** sample A-1, **c** sample A-2, and **d** NaCl (negative control) from 0 and 300 s

Figure 6 shows the blood vessel of the sample A-1 and A-2. All endpoints occurred in the positive control group starting from 0 s. The HET-CAM effects are scored and classified to give risk assessments analogous to those of the Draize rabbit-eye test. Previous studies have reported a good correlation (76%) between in vitro tests with HET-CAM and in vivo tests with the Draize method (including electron microscopy) [22, 30]. The test material groups did not show the presence of endpoints and had similar responses with the negative control. Therefore, based on the results, it was found that the samples did not irritate the hen’s egg membrane. It can be concluded that the PMHS samples were in vitro non-toxic as well as PDMS [31]. The similarity of PMHS characteristics with commercial PDMS confirms that PMHS can be applied as a new alternative material as a substitute for vitreous humour.

## Conclusion

Low- and medium-viscosity PMHS with suitable properties as vitreous humour substitution has been synthesized from DCMS through the hydrolysis-condensation method using DE and DCM solvent. The use of basic solvents and setting up the temperature in the condensation process can increase the viscosity. PMHS samples had a lower refractive index value than PDMS but were still in the range of vitreous humour substitution material requirement. All samples had a transparent physical appearance and had a typical functional groups of PMHS. Both low- and medium-viscosity PMHS samples were non-toxic through the in vitro HET-CAM test. These results provide evidence that PMHS can be considered as a new alternative vitreous humour substitution material.

## Data Availability

No datasets were generated or analysed during the current study.
